# Cytocompatible Biocatalyzed Surface-Initiated PhotoATRP Mediated by Red Light Irradiation in Open Air

**DOI:** 10.1021/jacsau.6c00580

**Published:** 2026-04-29

**Authors:** Yuwen Zhang, Elena Avanzini, Martina Ferrara, Rebecca Busetto, Veronica Torresan, Alessandro Gandin, Giovanna Brusatin, Cristian Pezzato, Francesca Lorandi, Edmondo M. Benetti

**Affiliations:** † Laboratory for Macromolecular and Organic Chemistry, Department of Chemical Sciences, 165501 University of Padova, via Marzolo 1, Padova 35131, Italy; ‡ Department of Industrial Engineering, University of Padova, via Marzolo 9, Padova 35131, Italy

**Keywords:** polymer brushes, controlled radical polymerization, photopolymerization, surface functionalization, protein adsorption

## Abstract

Surface-initiated atom transfer radical polymerization (SI-ATRP) enables the fabrication of functional polymer brushes, yet achieving simultaneous oxygen tolerance and controlling polymer growth and compatibility to biological environments remains challenging. Here, we report a hemoglobin (Hb)-catalyzed, red light-mediated SI-ATRP (SI-bioATRP) that proceeds efficiently in open air. In the presence of methylene blue (MB^+^) as a photosensitizer and trace dimethyl sulfoxide (DMSO) as an oxygen scavenger, red light excitation provides the rapid reduction of Hb­(Fe^III^) to Hb­(Fe^II^), which acts as activator for ATRP. This cooperative Hb/MB^+^/red light system promotes the controlled growth of chemically different brushes exceeding 300 nm in thickness, while analogous polymerizations in solution proceed in an uncontrolled manner. Mechanistic and calorimetric analyses reveal that the controlled behavior of SI-bioATRP arises from the intrinsic tendency of Hb to physisorb on initiator surfaces and dormant/propagating brush interfaces, ensuring an effective activation-deactivation equilibrium. The resulting process is fully oxygen-tolerant and cytocompatible and operates even in cell-culture media, enabling in situ polymer brush formation under biologically relevant conditions.

## Graphical abstract

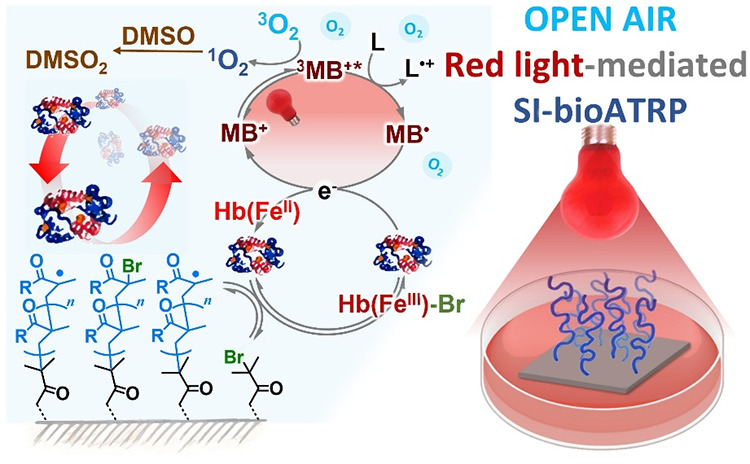

## Introduction

Surface-initiated atom transfer radical polymerization (SI-ATRP) has emerged as one of the most effective methods to generate polymer brushes of diverse composition from a variety of substrates.^1^ Some of the most recent developments in SI-ATRP closely followed the progressive advances achieved in the corresponding solution ATRP methods. These include the use of reducing agents^2–5^ and the application of light in conjunction with electron donors^6–8^ for catalyst regeneration.

However, enabling the large-scale modification of different substrates and biomaterials through SI-ATRP requires addressing several standing challenges.^9^ These included (i) the capability of modifying large surfaces, (ii) the need for oxygen-tolerant processes, and (iii) the development of nontoxic catalytic systems.

In search of polymer grafting processes compatible with biomaterials’ modification, a few years ago, we turned our attention to hemoglobin (Hb) as a biocatalyst for the synthesis of polymer brushes through surface-initiated biocatalyzed ATRP (SI-bioATRP).^10^

Similar to other metalloproteins, Hb features metal centers capable of catalyzing the homolytic cleavage of C–Br bonds to generate carbon-centered radicals that can subsequently trigger polymer chain growth in the presence of vinyl monomers through ATRP.^11–13^

Our initial studies on SI-bioATRP, however, suggested that polymer brush growth could be triggered just when Hb is “forced” to interact with an initiator-functionalized surface through physisorption, *i.e.*, when proteins adsorb on the surface while altering their structure,^14,15^ whereas just limited brush thicknesses could be obtained.^10^ In addition, a very large excess (≥200 equiv relative to Hb) of sodium ascorbate (NaAsc) was applied to reduce Hb­(Fe^III^)—present in commercial, lyophilized methemoglobin—to Hb­(Fe^II^), which is required for activating ATRP initiators. Finally, SI-bioATRP was very sensitive to oxygen, enabling the growth of brushes just under inert conditions. While these experimental settings are still adopted in recent studies on SI-bioATRP,^16,17^ they might simultaneously limit the applicability of SI-bioATRP within technologically relevant formulations.

More generally, very little has been explored about the factors that govern SI-bioATRP and the parameters that can be tuned for ensuring a rapid and controlled brush synthesis and for providing tolerance toward environmental conditions.

Motivated by the possibility of developing a biocatalyzed and cytocompatible, fully oxygen-tolerant process for polymer brush synthesis, here we demonstrate that opportune adjustment of reaction conditions gives access to a highly controlled SI-bioATRP process that is compatible with open-air setups.

An efficient biocatalyst activation emerges as the key to prompt brush growth. In this work, we bring evidence that the application of red light irradiation in the presence of methylene blue (MB^+^) as photosensitizer enables the fast reduction of Hb­(Fe^III^) to Hb­(Fe^II^), effectively providing the activator species for the SI-bioATRP process. In a similar way as recently described for Cu-based catalytic systems applied for solution polymerization^7^ as well as for brush synthesis,^18^ red light excitation of MB^+^ generates the triplet-state ^3^MB^+^*, which is reduced by an electron donor to MB^•^ (Figure 1). The latter reduces Hb­(Fe^III^) species to Hb­(Fe^II^) activators, simultaneously reforming ground-state MB^+^.

**Figure 1:**
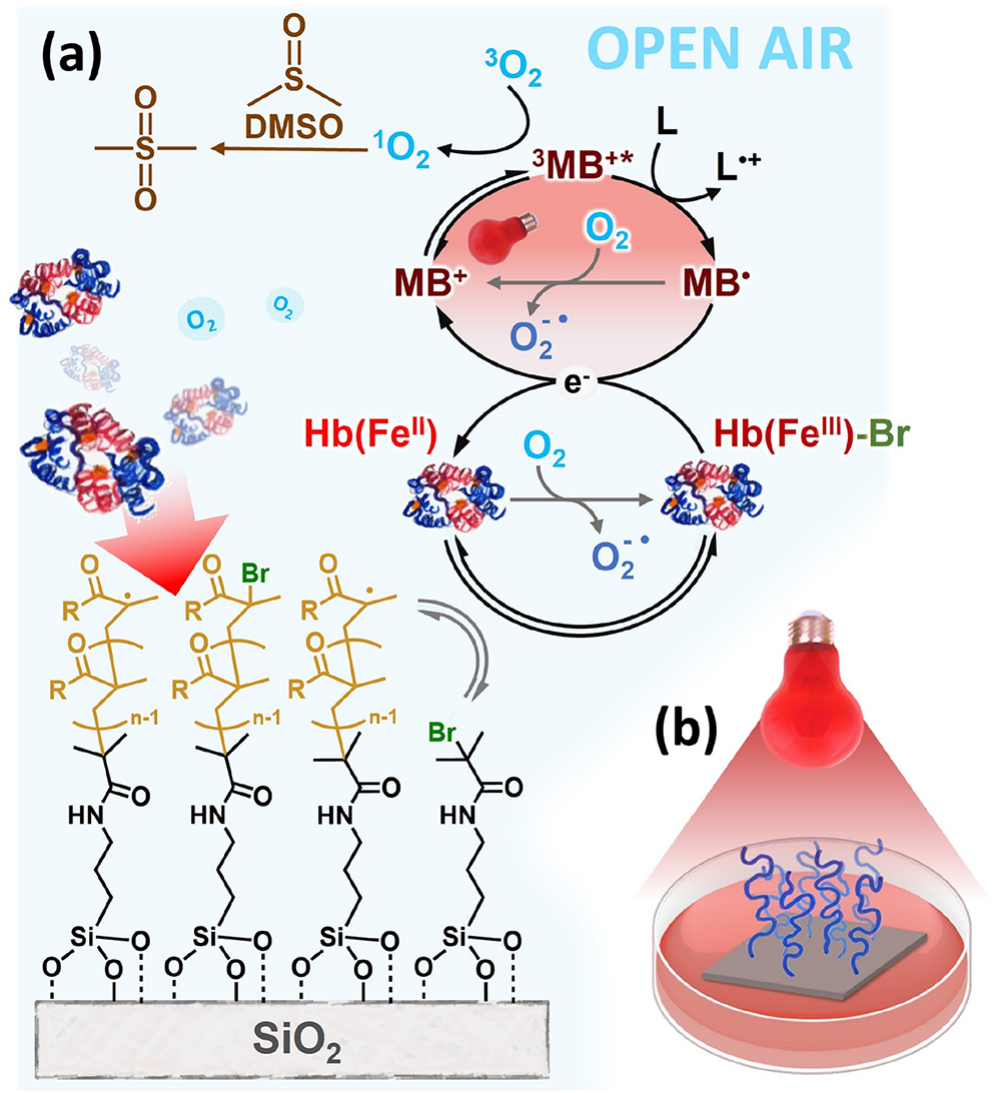
(a) The proposed mechanism of red light-mediated SI-bioATRP. Red light irradiation excites the ground-state MB^+^ to the singlet-state excited species that undergoes rapid intersystem crossing to generate the triplet-state ^3^MB^+*^. The latter is involved in a reductive quenching cycle, whereby an electron donor (*i.e.*, an excess of aliphatic amine) reduces ^3^MB^+*^ to the semireduced form MB^•^, which then reduces Hb­(Fe^III^) to Hb­(Fe^II^) that activates alkyl halide initiators or dormant chains. The reduction of Hb­(Fe^III^) results in the simultaneous regeneration of ground-state MB^+^. The formation of the Hb­(Fe^II^) activator through the photocatalytic cycle triggers the ATRP equilibrium when proteins interact with ATRP initiator-functionalized surfaces, enabling the growth of polymer brushes. At the same time, the triplet-state ^3^MB^+*^ is capable of reacting with triplet-state oxygen ^3^O_2_ dissolved in the reaction mixture. The reaction between ^3^MB^+*^ and ^3^O_2_ provides singlet oxygen, which recombines with DMSO forming dimethyl sulfone. (b) This process guarantees an efficient consumption of oxygen and enables the synthesis of thick brushes under open air.

Effective consumption of O_2_ is additionally attained by complementing the reaction mixture with relatively small amounts of dimethyl sulfoxide (DMSO). This reacts with singlet oxygen formed through the reaction between ^3^MB^+^* and ^3^O_2_, finally providing dimethyl.

We further emphasize that achieving controlled polymerization in red light-mediated SI-bioATRP critically relies on the intrinsic characteristics of the biocatalyst and the surface-confined nature of the process.

Hb is a tetrameric metalloprotein in which the Fe-coordinating heme groups are embedded within hydrophobic pockets,^19,20^ with only limited access to the Fe centers.^20–22^ While small alkyl halide initiators can readily reach these catalytic sites, we hypothesize that the approach of bulkier propagating chains is sterically hindered, thereby limiting the efficient activation-deactivation equilibrium required for controlled ATRP. Hence, in solution, access of bulky propagating chains to the partially buried heme centers is sterically hindered, limiting the effective catalyst-chain interactions required for ATRP equilibrium. As a result, while small initiators are readily activated, deactivation of growing chains is inefficient, leading to behavior resembling free-radical polymerization.

In contrast, an intimate contact between Hb and initiating/propagating interfaces is established during the corresponding surface-initiated process, due to the intrinsic tendency of biomolecules toward surface passivation through physisorption.^23,24^ This spontaneous process is surface-energy driven^25^ and could be effectively monitored through a combination of different surface-sensitive techniques, including quartz crystal microbalance with dissipation (QCM-D) and isothermal titration calorimetry (ITC).

The synergy between red light irradiation and MB^+^, enabling efficient catalyst activation, and the intrinsic affinity of Hb toward surface-immobilized brushes arises as critical determinants in achieving the fully oxygen-tolerant synthesis of thick (>200 nm) brushes through a cytocompatible grafting process.

## Results and Discussion

### Red Light-Mediated SI-bioATRP in Open Air

We initially highlighted the role of MB^+^ as a photosensitizer for red light-mediated SI-bioATRP. Previously reported bioATRP mixtures in solution^26^ and from surfaces^10^ exploited sodium ascorbate (NaAsc) for the reduction of Hb­(Fe^III^) to Hb­(Fe^II^), the latter acting as activator for polymerization. However, a very large excess of reducing agent was necessary to attain a sufficient content of Hb­(Fe^II^) for starting polymer growth. Reduction of methemoglobin by ascorbic acid under anaerobic conditions was reported to proceed through a first rapid phase, followed by a second phase that is ∼10 times slower.^27,28^ During the first phase, only ferric centers in β chains are reduced, whereas the reduction of Fe^III^ in α chains is much slower and it eventually competes with the tendency of Hb to undergo autoxidation if oxygen traces are present. This behavior highlights that heterogeneities within the protein configuration affect its reactivity, while suggesting that more efficient reduction pathways may be identified. In the present study, we explored an alternative system based on MB^+^ coupled with red light irradiation, which has already proven to enable the prompt and efficient activation of Cu catalysts during red light-mediated photoATRP.^18^

Relevantly, MB^+^ has been extensively applied as a therapeutic agent for methemoglobinemia,^29^ to reduce Hb­(Fe^III^) to Hb­(Fe^II^) under physiological conditions by exploiting electron transfer from NAD­(P)­H. Indeed, cyclic voltammetry (*C–V*) of MB^+^ in acetate buffer at pH 6 showed that the introduction of Hb caused an enhancement in the cathodic current peak—attributed to the reduction of the dye—and a decrease in the corresponding oxidation peak (Figure S1). This indicates that once the reduced forms of MB^+^ (*i.e.*, MB^•^ and leucomethylene blue, LMB)^30^ are generated near the electrode surface, they can reduce Hb­(Fe^III^) to Hb­(Fe^II^), regenerating MB^+^ that is immediately reduced again, thus producing a catalytic signal.

Similarly, the irradiation of MB^+^ with red light in the presence of an electron donor generates MB^•^. The latter has a standard reduction potential *E* ^θ^(MB^•^/LMB) −0.3 V *vs* saturated calomel electrode (SCE)^31^ and therefore is likely capable of reducing Hb­(Fe^III^) to Hb­(Fe^II^) (*E* ^θ^ = −0.33 V *vs* SCE).^32^ Hence, MB^+^ together with an electron donor and red light could be exploited to generate Hb­(Fe^II^), which then reacts with alkyl halides to form propagating radicals.

*C–V* of Hb adsorbed onto a glassy carbon electrode showed a catalytic response upon addition of increasing amounts of a water-soluble alkyl bromide, hydroxy-2-ethyl-2-bromoisobutyrate (HEBiB)—commonly used as ATRP initiator—confirming that Hb­(Fe^III^) can reductively cleave the C–Br bond of HEBiB (Figure S3). The subsequent addition of MB^+^ resulted in a further enhancement of the cathodic current corresponding to the reduction of adsorbed Hb­(Fe^III^). Moreover, further addition of HEBiB led to an increase in the current values corresponding to the reduction of both MB^+^ and Hb­(Fe^III^). These observations indicate that upon electrochemical reduction, the dye can reduce Hb­(Fe^III^) to Hb­(Fe^II^), which in turn activates HEBiB.

Hence, we hypothesized that the MB^+^/Hb system under red light irradiation can trigger polymer brush growth through activation of surface-tethered alkyl bromide initiators/chain ends (Figure 1a), analogously to the previously reported MB^+^/Cu system.^7^ Noteworthy, the use of MB^+^ in conjunction with red light irradiation and relatively low amounts of DMSO provides tolerance toward oxygen within the polymerization system, enabling the growth of brushes under environmental conditions. This occurs when triplet-state ^3^MB^+^* reacts with ^3^O_2_ yielding singlet oxygen that quickly recombines with DMSO to produce the corresponding sulfone (Figure 1a).^18^

Red light-mediated SI-bioATRP was initially tested in a fully open air setup (Figure 1b), using an ATRP initiator-functionalized SiO_2_ substrate deposited on a Petri dish which was filled by a polymerization mixture comprising oligo­(ethylene glycol) methacrylate (OEGMA) (20 vol % in acetate buffer and 10 vol % DMSO), Hb (16 μM) as catalyst and MB^+^ (0.25 mM) as photosensitizer, *N,N,N,N,N*-pentamethyldiethylenetriamine (PMDETA, 6 mM) as electron donor, and adjusting the pH of the mixture to 6.0. Red light irradiation (λ_max_ = 625 nm, 4 mW cm^–2^) triggered the growth of uniform poly­[(oligoethylene glycol)­methacrylate] (POEGMA) brushes, as it was recorded by *ex situ* variable angle spectroscopic ellipsometry (VASE) (Figure 2a). After 30 min of reaction, a dry thickness (*T*_dry_) of 11.9 ± 0.4 nm was reached, while brush thickening progressively continued, until reaching a *T*_dry_ of 25.8 ± 0.6 nm following 90 min of red light irradiation and 32.2 ± 0.4 nm after 270 min.

**Figure 2:**
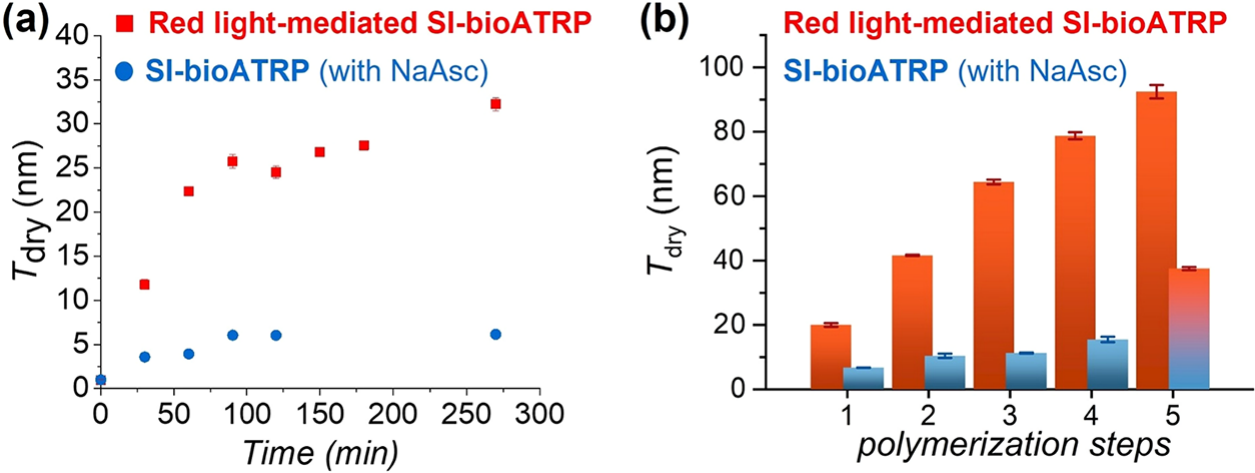
(a) Dry thickness (*T*_dry_) of POEGMA brushes recorded by ex situ VASE, following red light-mediated SI-bioATRP (red squares) and NaAsc-mediated SI-ATRP (blue dots) over time. (b) Dry thickness of POEGMA brushes measured after 90 min of 5 consecutive red light-mediated SI-bioATRP steps on the same substrate (red bars), and 4 consecutive NaAsc-mediated SI-bioATRP steps followed by a 5^th^ step via red light-mediated SI-bioATRP on the same substrate (blue and blue-red bars). General conditions: OEGMA 20 vol % in acetate buffer, [Hb] = 16 μm. Conditions for red light-mediated SI-bioATRP: DMSO 10 vol %, [MB^+^]:[Hb]:[PMDETA] = 15.6:1:375, λ_max_ = 625 mM, intensity = 4 mW cm^–2^, open air. Conditions for NaAsc-mediated SI-bioATRP:[NaAsc]:[Hb] = 275:1, deoxygenated solution.

Relevantly, MB^+^, Hb, and red light irradiation were essential for the growth of POEGMA brushes, as demonstrated by control experiments, where the absence of one of these components during polymerization resulted in negligible polymer growth from the surface (Table S1). When PMDETA was not present, brush growth was observed, the polymer films showed poor uniformity and inconsistent results were obtained upon repeated experiments. We speculate that some functional groups within the protein structure can donate electrons to the excited-state ^3^MB^+^* promoting brush growth. Therefore, to ensure reproducible results, PMDETA was always introduced in the polymerization mixtures as an electron donor.

The growth of POEGMA brushes through red light-mediated SI-bioATRP was additionally compared with the corresponding SI-bioATRP process exploiting NaAsc as a reducing agent. In this case, the polymerization mixture comprised OEGMA (20 vol % in acetate buffer), Hb (16 μM), and NaAsc (4.4 mM). The final pH of the solution was 5.5, and it was subjected to 30 min of Ar bubbling prior to exposure to ATRP initiator-functionalized SiO_*x*_ substrates. As shown in Figure 2a, during SI-bioATRP mediated by NaAsc, POEGMA brush thickness increased slowly during the first 2 h of polymerization, until a plateau was reached when *T*_dry_ leveled at ∼6 nm.

Overall thicker brushes could be obtained through SI-bioATRP processes by performing multiple reinitiation steps from the same substrates. As was previously demonstrated, SI-bioATRP requires a renewal of the biocatalyst layer at the grafted-chain interface in order to attain prompt brush growth. This can be accomplished by reinitiating the grafting process through the application of a new reaction mixture, which promotes refreshing of the Hb layer that spontaneously forms on the brush interface.^10^ In line with this previous observation, by subjecting ATRP initiator-functionalized substrates to four sequential NaAsc-mediated SI-bioATRP steps, each of them including a 90 min incubation in the polymerization mixture, a final *T*_dry_ of 16 ± 1 nm (Figure 2b) was reached. However, multiple reinitiation via the corresponding red light-mediated SI-bioATRP process provided significantly higher brush thickenings, with *T*_dry_ progressively increasing after each reinitiation cycle and reaching 92 ± 2 nm after five subsequent 90 min-steps.

The substantially higher activity of the biocatalytic system generated through MB^+^/red light irradiation could be clearly highlighted by reinitiating polymerization from a POEGMA brush substrate previously fabricated via 4 reinitiation cycles performed by NaAsc-mediated SI-bioATRP (Figure 2b). Irradiating with red light a 16 ± 1 nm thick POEGMA brush immersed in a reaction mixture where NaAsc was replaced by 0.25 mM MB^+^, a significant increment in film thickness was measured, with *T*_dry_ reaching 38 ± 1 nm after 90 min of reaction. This result also confirmed that the relatively slower brush growth recorded during NaAsc-mediated SI-bioATRP was not due to irreversible chain termination, but rather, it was correlated to a comparatively lower activity of the biocatalytic system, *i.e.*, to a lower content of Hb­(Fe^II^) activators.

In order to confirm this assumption, we monitored the reduction of Hb­(Fe^III^) to Hb­(Fe^II^) by UV-vis spectroscopy while focusing on the intensity of the absorption band centered at 404 nm (Figure 3a,b), *i.e.*, the Soret band in methemoglobin arising from a π–π* transition within the porphyrin rings.^33^

**Figure 3:**
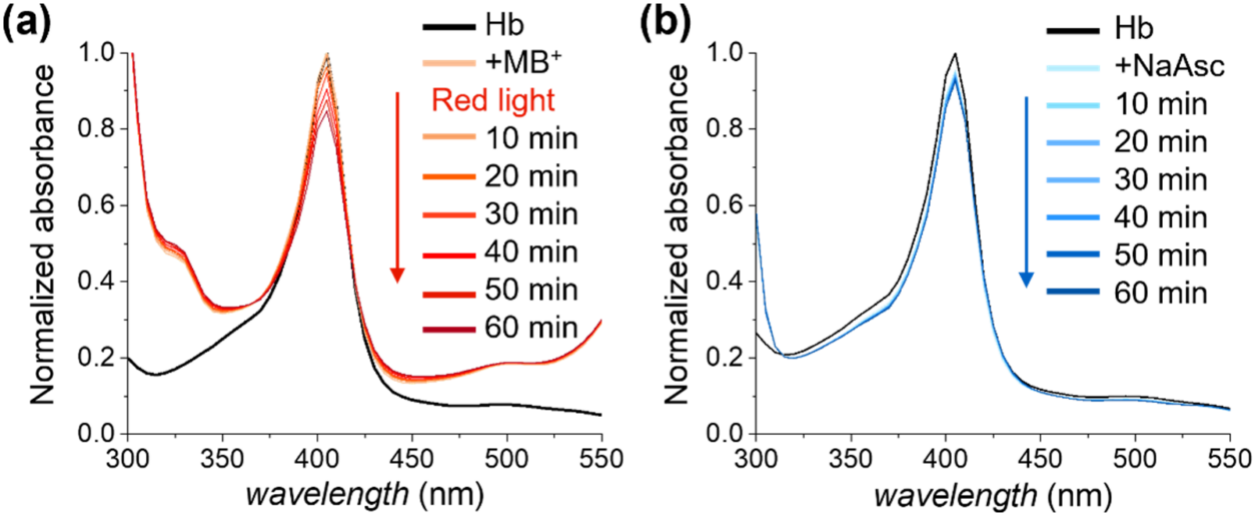
UV-vis spectra of Hb in acetate buffer at pH 6, in the presence of OEGMA (0.108 M) and MB^+^ under red light irradiation (λ_max_ = 625 mM, intensity = 4 mW cm^–2^), [OEGMA]:[MB^+^]:[Hb]:[PMDETA] = 27000:15.6:1:375, 2.5 vol % DMSO, open air (a); NaAsc, [OEGMA]:[NaAsc]:[Hb] = 27000:275:1, deoxygenated setup (b).

Addition of MB^+^ to a typical polymerization solution of OEGMA in acetate buffer comprising Hb and PMDETA (so that the molar ratio [MB^+^]:[Hb]:[PMDETA] = 15.6:1:375) was mirrored by the appearance of different adsorption bands between 300 and 340 and 450-510 nm, which are characteristic of MB^+^. However, no detectable decrease in absorbance was recorded for the band centered at 404 nm, indicating that the presence of MB^+^ alone does not trigger the reduction of Hb­(Fe^III^). The solution was subsequently irradiated with red light, and UV-vis spectra were recorded every 10 min. A prompt and progressive decrease in the absorbance at 404 nm suggested a gradual reduction of Hb­(Fe^III^) to Hb­(Fe^II^), confirming efficient activation of the biocatalyst when the polymerization solution was exposed to red light (Figure 1).

When NaAsc was added to a similar polymerization solution including OEGMA in acetate buffer and Hb, with a molar ratio [NaAsc]:[Hb] of 275:1 as in a traditional SI-bioATRP, a different scenario was highlighted through UV-vis. Only a slight initial decrease of the absorbance at 404 nm upon addition of NaAsc could be recorded, which was not followed by a significant decrease after 60 min of incubation (Figure 3b). The observed rate constant (*k*_obs_) for the reduction of Hb­(Fe^III^) was determined to be 5.2 × 10^–3^ s^–1^ for the system containing MB^+^ under red light irradiation, whereas a 5-fold lower *k*_obs_ value (0.9 × 10^–3^ s^–1^) was calculated when NaAsc was employed as reductant (Figure S4). Hence, the use of MB^+^ in conjunction with red light provides a faster reduction of Hb­(Fe^III^) in comparison with NaAsc, resulting in more rapid brush growth from ATRP initiator-functionalized substrates.

The extremely efficient biocatalyst activation enabled by red light irradiation/MB^+^ thus allowed us to fabricate very thick brush coatings under completely open-air conditions. As highlighted in Figure 4, polymer brush growth could be reinitiated through 14 sequential polymerization steps, reaching a *T*_dry_ of ∼300 nm, and confirming the very controlled nature of red light-mediated SI-bioATRP. Relevantly, such high values of brush thickness were rarely reported in the literature for an RDRP process^34^—especially for oxygen-tolerant polymerizations—whereas they are unprecedented for biocatalyzed chain-growth polymerizations.

**Figure 4:**
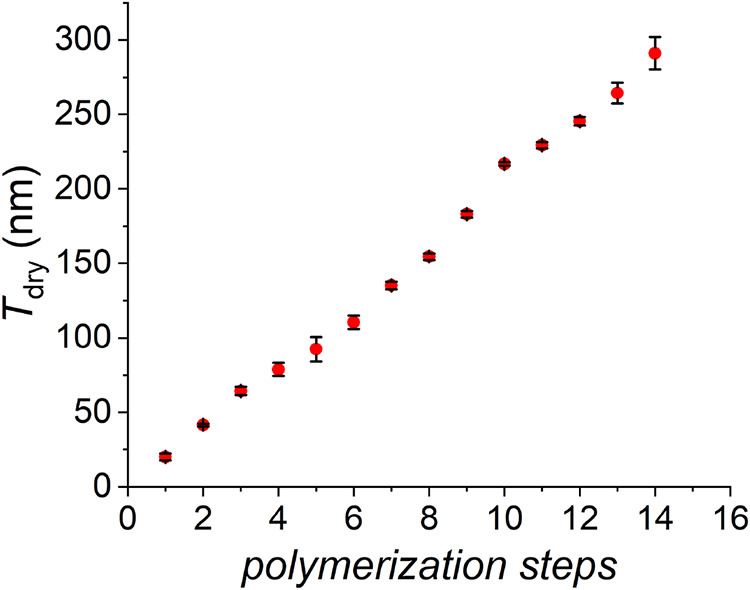
Dry thickness of POEGMA brushes measured after 90 min of irradiation for 14 consecutive polymerization steps on the same substrate, in open air. Conditions: OEGMA 20 vol % in acetate buffer with DMSO 10 vol %, [Hb] = 16 μM, [MB^+^]:[Hb]:[PMDETA] = 15.6:1:375, λ_max_ = 625 mM (4 mW cm^–2^).

### Red Light-Mediated bioATRP in Solution

The Hb/MB^+^-catalyzed photoATRP was further evaluated in a homogeneous solution. A polymerization mixture containing HEBiB ([OEGMA]:[HEBiB] = 100:1) was prepared, sealed in a vial, and irradiated with red light (λ_max_ = 625 mM, 4 mW cm^–2^) under an Ar atmosphere for the desired duration (Figure S5–S6). At pH 6, *i.e.*, the same pH used for surface-initiated polymerization, no polymer formation was observed, although the reason for this remains unclear. When the reaction was carried out at pH 7, polymerization proceeded, yielding detectable polymers. However, the resulting polymer showed *M_n_* values measured by SEC significantly exceeding the theoretical values, and it exhibited consistently high dispersity (*Đ*), indicating poor control over the polymerization process in solution (Figures S5 and S6). Solution polymerizations at pH 6 and 7 were then repeated in open air. No polymer was formed at pH 6, whereas polymerization occurred at pH 7. However, the obtained polymer was highly viscous and likely cross-linked, as it could not be solubilized and analyzed by SEC.

Red light-mediated bioATRP in deoxygenated solutions was also conducted across a pH range from 4 to 9. No polymer formation was observed for pH ≤ 6, and a minimum pH of 7 was required for polymer formation (Table S2). Although polymerization occurred at pH 7, *C–V* studies revealed that the redox activity of the Hb/MB^+^ system toward alkyl halide was not substantially different at pH 7 and 6, as similar cathodic currents were recorded under both conditions (Figure S3). Thus, the influence of pH on the reactivity of the Hb/MB^+^ system can be ruled out to explain the lack of polymerization at pH < 7.

At the same time, even if polymerization occurred at pH > 7, the resulting polymer showed high *Đ* and approximately constant values of *M_n_* with increasing monomer conversion. These trends are consistent with free (uncontrolled) radical polymerization (FRP), suggesting that while the Fe centers within the protein can activate the alkyl halide initiators, they are unable to efficiently deactivate the bulkier propagating macroradicals.

### Interaction of Hb and POEGMA Brush Interfaces

Despite red light-mediated bioATRP in solution showed traits that are characteristic of uncontrolled FRP, we hereby demonstrated that the corresponding surface-initiated process proceeded in a controlled manner, enabling the synthesis of thick brushes and multiple chain extensions.

We believe that this was due to the intrinsic tendency of Hb to physisorb on initiator-bearing surfaces and propagating/dormant brush interfaces, a phenomenon that leads to partial protein unfolding,^15,24^ and thus to an increased exposure of heme/hemin groups providing not only activation of initiator/dormant chains but also their more effective deactivation (according to the ATRP equilibrium).

Protein physisorption on brushes is often unavoidable and takes place through different mechanisms involving penetration through the brush (primary and tertiary adsorption), or interaction with the brush outer surface (secondary adsorption).^35,36^

Here, we exploited isothermal titration calorimetry (ITC) and quartz crystal microbalance with dissipation (QCM-D) to quantitatively evaluate the interaction between Hb and the initiator/brush interfaces and compare it with the case where dormant polymers in solution are incubated with the protein biocatalyst.

In particular, a dispersion of 6 mg mL^–1^ 200 nm diameter SiO_2_ nanoparticles (NPs) decorated with POEGMA brushes (previously synthesized by SI-ARGET ATRP, Supporting Information) were titrated with Hb solution while recording the heat transfer associated with the isothermal titration.^37–39^ A similar setup was simultaneously applied in the case of free POEGMA chains synthesized via SI-ARGET ATRP, and subjected to Hb titration (the concentrations of SiO_2_-grafted and free POEGMA chains were kept nearly constant).

A clear interaction was recorded between Hb and SiO_2_-*g*-POEGMA (Figure 5a), as evidenced by the significantly higher heat of injection recorded upon the first few additions of Hb solution (red signals in Figure 5a), in comparison with the corresponding heat of dilution measured for the same dispersion of SiO_2_-*g*-POEGMA NPs (blue signals in Figure 5a). The recorded heat of injection decreased upon each subsequent Hb addition, likely due to the progressive saturation of nonspecific binding sites present on the POEGMA grafts and the concomitant formation of the protein corona (Figure 5b). In contrast, the heat of injection recorded when Hb was added to free POEGMA chains in solution (red signals in Figure 5c) was comparable and nearly overlapped with the heat of dilution (blue signals in Figure 5c). This indicated that Hb did not appreciably interact with POEGMA chains when these are not grafted to a solid surface to form a brush interface (Figure 5d). The correlation between the enthalpy of interaction and the molar ratio of Hb and POEGMA (Figure S8) could be used to determine the number of binding sites (*n*) and the dissociation constant (*K*_D_) for POEGMA brushes grafted on SiO_2_ NPs. This provided *n* ∼ 10—suggesting multiple binding of Hb to each POEGMA graft on the surface—and *K*_D_ = (3.1 ± 1.0) × 10^–6^ M (and thus a corresponding association constant *K*_A_ = (3.2 ± 1.0) × 10^5^ M^–1^).^40^ The enthalpy associated with the formation of Hb corona on POEGMA brushes could be estimated as Δ*H* = −12.13 ± 1.3 kJ mol^–1^, which quantified the exothermic, hydrophobic interactions that drive the adsorption of Hb proteins on and within the POEGMA brush layer.^41,42^

**Figure 5:**
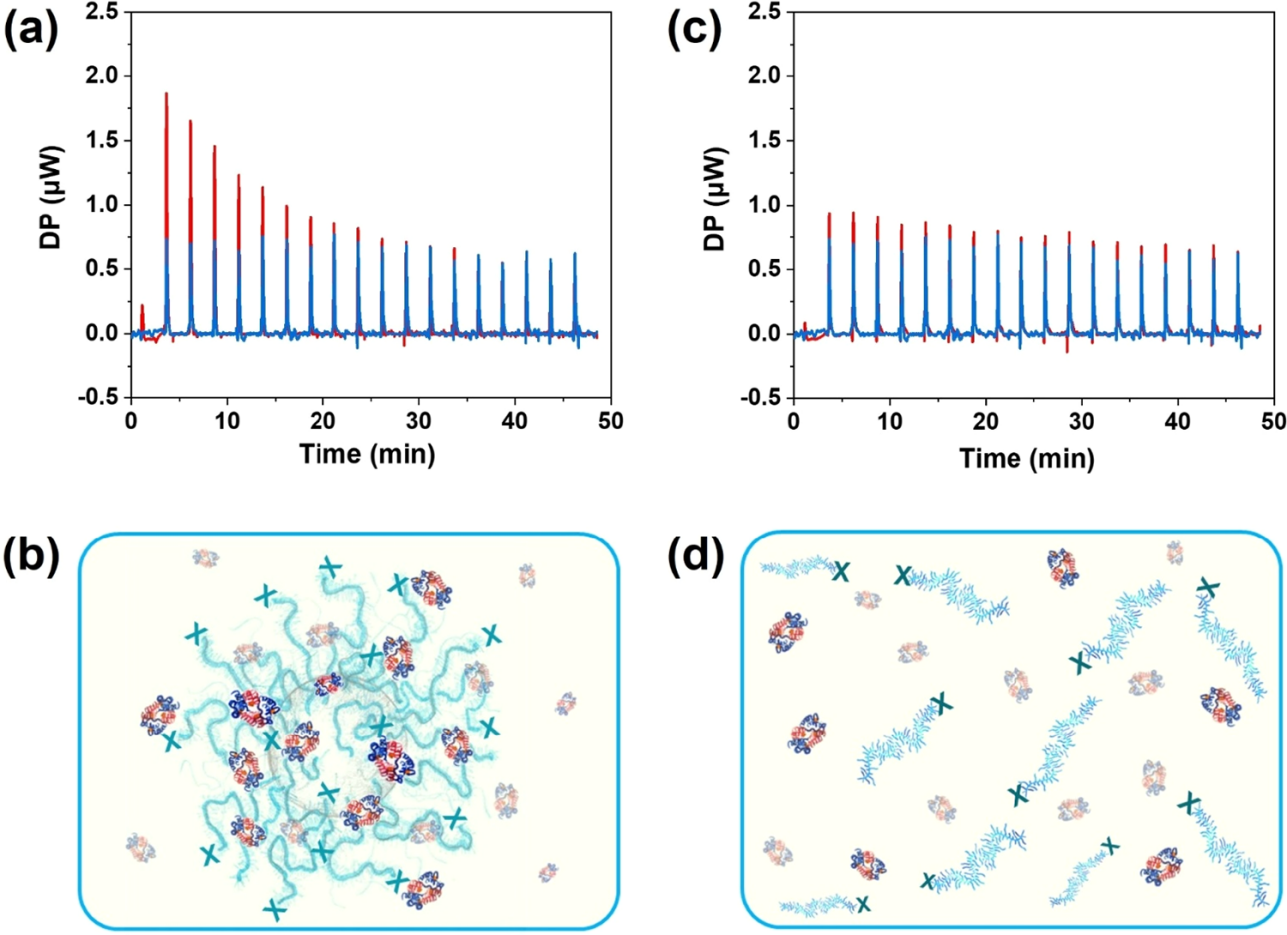
(a) ITC measurements performed by titrating with increasing amounts of Hb dispersions of SiO_2_ NPs functionalized with POEGMA brushes. (b) The formation of Hb corona could be inferred from ITC measurements. (c) ITC measurements performed on free POEGMA chains subjected to sequential injections of Hb, which indicated that (d) no measurable interactions could be recorded between the biocatalyst and polymer chains in solution. The heat of dilution (blue signals in (a) and (c)) was obtained by recording the values of differential power (DP) after sequential injections of 2 μL aliquots of acetate, whereas interaction between brushes/free polymer and Hb (red signals in (a) and (c)) was recorded by sequential injection of 2 μL aliquots of Hb solutions (600 μM) in acetate buffer into 0.07 nM dispersions of POEGMA brush-functionalized SiO_2_ NPs and 1.8 μM solutions of free POEGMA in acetate buffer.

The interaction between Hb and POEGMA brushes grown from macroscopic substrates was additionally evaluated by QCM-D. SiO_2_-coated QCM-D sensors were initially functionalized with ATRP initiator and later on subjected to red light-mediated SI-bioATRP of OEGMA for 40 min (Figure 6a), obtaining a POEGMA brush layer with *T*_dry_ of ∼20 nm. Hb interaction was evaluated on both ATRP initiator-functionalized sensors and POEGMA brush-decorated counterparts. Both sensor types were initially exposed to a solution comprising all components of the polymerization mixture except for Hb (i.e., 20 vol % OEGMA in acetate buffer with 10 vol % DMSO, 0.25 mM MB^+^, 6 mM PMDETA) (Section 2, Figure 6b,c and S8). Subsequent injection of an identical mixture, although complemented with 16 μM Hb, caused a clear decrease in Δ*F*, which was correlated to an increment in hydrated mass (Δ*m*_(Hb)_) from the formation of a protein layer on the functionalized sensors. A larger shift of Δ*F* was measured for ATRP initiator-functionalized sensors compared with POEGMA brush-decorated surfaces, highlighting how the relatively hydrophobic ATRP initiator self-assembled monolayers (SAMs) attracted a relatively larger mass of proteins. However, a clear adsorption of Hb was also recorded on POEGMA brushes, corroborating ITC analysis.

**Figure 6:**
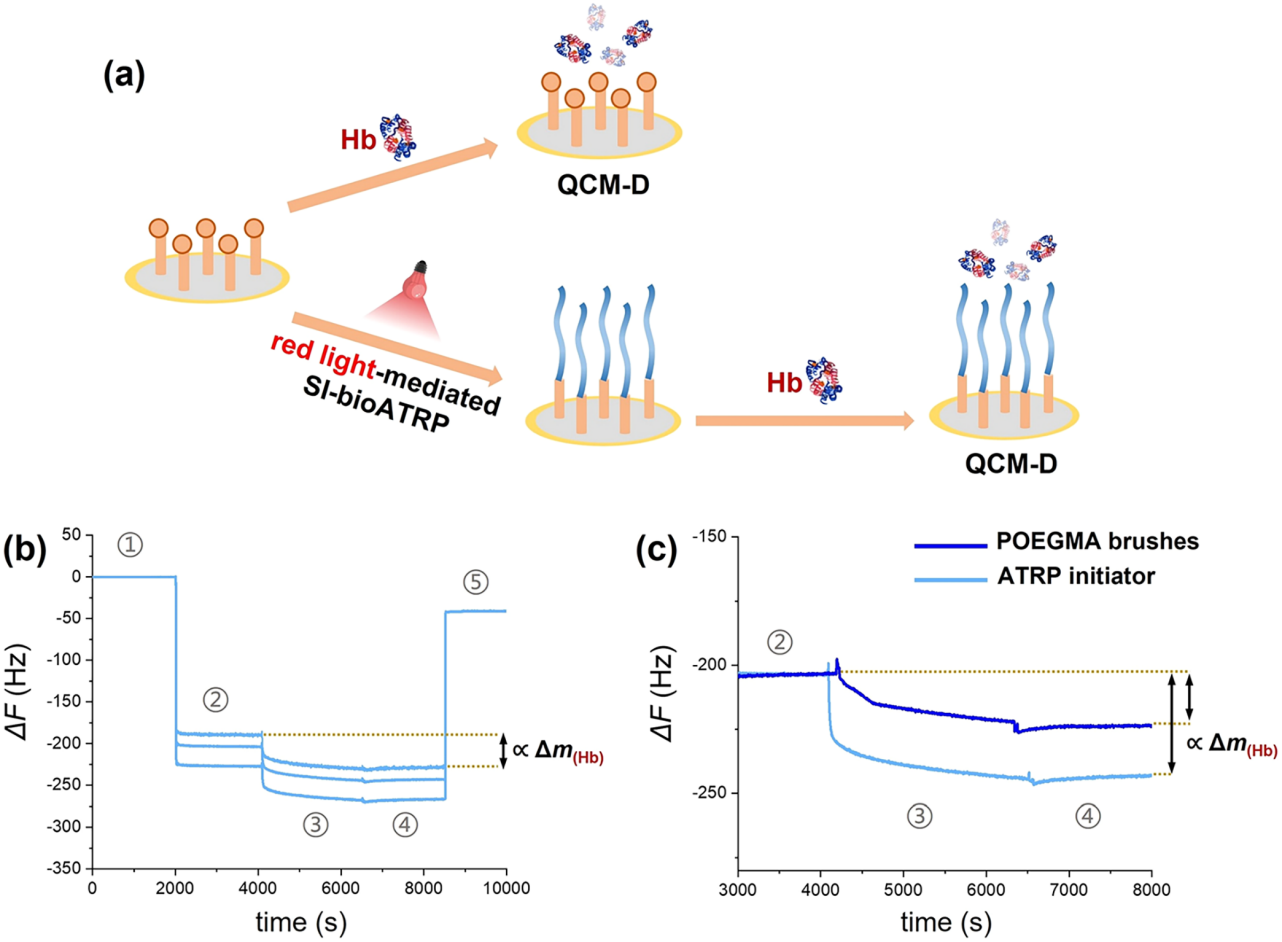
(a) QCM-D sensors were functionalized *ex situ* with ATRP initiator and POEGMA brushes by red light-mediated SI-bioATRP. QCM-D sensograms displayed the variation of Δ*F* (for three different overtones: *f*-5th, *f*-7th, and *f*-9th) when (b) ATRP initiator-functionalized sensors and (c) POEGMA brush-bearing analogues were subjected to different mixtures. In both cases, the sensors were incubated with (1) ultrapure water, (2) polymerization mixture without Hb, (3) complete polymerization mixture including Hb, (4) Hb-free polymerization mixture, and (5) finally ultrapure water. The shift in Δ*F* between (2) and (4) was proportional to the increment in hydrated mass generated by protein physisorption.

These results confirm that the interaction between Hb and functionalized surfaces presenting initiator/dormant polymer chains always takes place, to an extent that is correlated to the relative affinity toward proteins by the interface. Intimate contact between propagating grafts and biocatalyst guarantees a much more efficient deactivation during red light-mediated SI-bioATRP, eventually gaining the control that is largely lost when such a polymerization process is performed in solution.

### Monomer Scope and Irradiation Wavelengths

Red light-mediated SI-bioATRP was applied to different monomers to demonstrate the compatibility of this process toward a variety of chemistries. Poly­[oligo­(ethylene glycol)­methyl ether acrylate] (POEGA) brushes with *T*_dry_ = 21.3 ± 0.6 were obtained after 90 min of reaction, thus reaching thicknesses comparable to those obtained by polymerizing the corresponding OEGMA for a similar time (Table 1, entries 1–2). Red light-mediated SI-bioATRP was also compatible with charged, zwitterionic, and acrylamide monomers (Table 1, entries 4–7), although in the cases of zwitterionic species, the obtained *T*_dry_ values were significantly lower compared to those obtained for neutral poly­(meth)­acrylates, presumably due to the marked resistance toward protein adsorption by the corresponding brushes.^43–45^

**Table 1:** Red Light-Mediated SI-bioATRP of Various Monomers in Open Air^a^

**entry**	**monomer**	***T***_**dry**_ **(nm)** ^b^
1	OEGMA	25.8 ± 0.8
2	OEGA	21.3 ± 0.3
3	SPMA	6.4 ± 0.5
4	METAC	8.1 ± 1.4
5	NIPAM	4.1 ± 0.4
6	MPC	3.7 ± 0.5

a Conditions: Monomer 20 vol % in acetate buffer with 10 vol % DMSO, pH = 6, [Hb] = 16 μm, [MB^+^]:[Hb]:[PMDETA] = 15.6:1:375, irradiated for 90 min with red light (λ_max_ = 625 nm, intensity = 4 mW cm^–2^).

b Measured by VASE. SPMA: 3-sulfopropyl methacrylate potassium salt; METAC: [2-(methacryloyloxy)­ethyl]­trimethylammonium chloride; NIPAM: *N*-isopropylacrylamide; MPC: 2-methacryloyloxyethyl phosphorylcholine.

The broad range of light absorption of MB^+^ ^18^ provides the opportunity for employing different light wavelengths to trigger the SI-bioATRP process (Figure 7). POEGMA brushes with *T*_dry_ ∼ 13 nm (Figure 7 and Table S3) were obtained upon 90 min of exposure to either UV or blue light (λ_max_ = 365 and 420 nm, respectively, keeping the intensity constant at 4 mW cm^–2^). POEGMA brushes with *T*_dry_ > 20 nm were achieved under exposure to λ_max_ = 475 and 565 nm. Finally, slower but appreciable brush growth was also recorded by subjecting the polymerization setup to NIR light (λ_max_ = 780 nm) irradiation.

**Figure 7:**
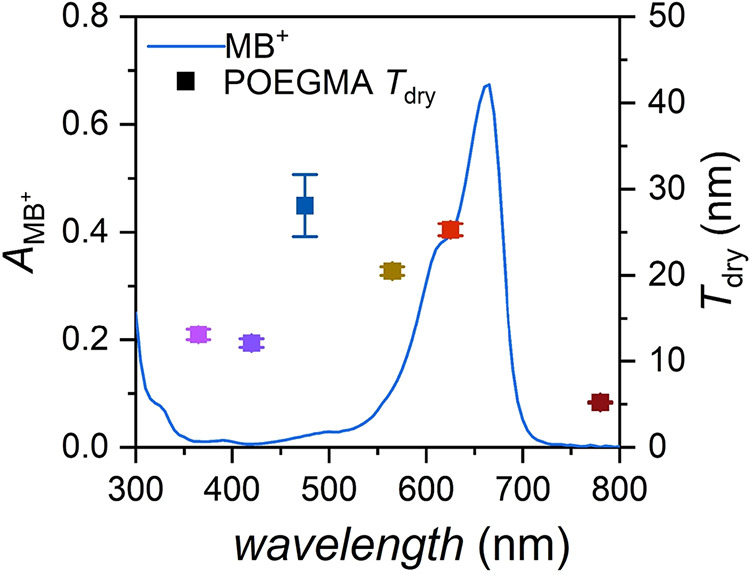
UV-vis spectrum of MB^+^ (blue line) and dry thickness of POEGMA brushes as a function of the applied light wavelength (squares). Conditions: OEGMA 20 vol % in acetate buffer with 10 vol % DMSO, pH = 6, [Hb] = 16 μm, [MB^+^]:[Hb]:[PMDETA] = 15.6:1:375, irradiated for 90 min under a light intensity of 4 mW cm^–2^ in open air.

### Cytocompatibility of Red Light-Mediated SI-bioATRP

The combination of Hb as a biocatalyst with the biocompatibility of MB^+^ and red light prompted us to test the applicability of red light-mediated SI-bioATRP within cell cultures. At first, we studied the growth of POEGMA brushes by replacing the acetate buffer with Dulbecco’s Modified Eagle’s Medium (DMEM) as a solvent. Within 90 min of irradiation, a polymer brush layer with *T*_dry_ = 10.6 ± 1.3 nm was obtained, which raised to *T*_dry_ = 23.1 ± 0.6 upon decreasing [MB^+^] from 0.25 to 0.1 mM. Then, we replaced the macromonomer OEGMA (*M_n_* ∼500 g mol^–1^, OEGMA_500_ in Table 2) with the higher-molar-mass analogue OEGMA_950_ (*M_n_* ∼ 950 g mol^–1^) since the lower-molar-mass OEGMA_500_ could comprise cell proliferation.^46^ At the same time, we reasoned that various components present in the DMEM, such as some amino acids, vitamins, or glucose, could act as electron donors for the (re)­generation of MB^•^ from the excited-state ^3^MB*. Thus, red light-mediated SI-bioATRP of OEGMA_950_ was performed in the absence of PMDETA, providing polymer brushes with *T*_dry_ = 6.6 ± 0.6 nm (Table 2, entry 3). Increasing [Hb] up to 20 times resulted in a modest increase in the thickness of the polymer layer, which diminished again when the amount of DMSO was decreased to 1 vol % to further improve the biocompatibility of the system (Table 2, entries 4–5). Nevertheless, by using an equimolar amount of Hb and MB^+^, POEGMA_950_ brushes with *T*_dry_ = 16 ± 2 nm were obtained within 90 min of red light irradiation in the absence of added electron donor and with only 1 vol % DMSO (Table 2, entry 7).

**Table 2:** Enhancing the Cytocompatibility of Red Light-Mediated SI-bioATRP in Open Air^a^

entry	monomer^b^	DMSO (vol %)	Hb (μM)	MB^+^ (mM)	electron donor	*T*_dry_ (nm)^c^
1	OEGMA_500_	10	16	0.25	PMDETA^d^	10.6 ± 1.3
2	OEGMA_500_	10	16	0.1	PMDETA^d^	23.1 ± 0.6
3	OEGMA_950_	10	16	0.1		6.6 ± 0.6
4	OEGMA_950_	10	80	0.1		13.4 ± 0.5
5^e^	OEGMA_950_	10	320	0.1		8.4 ± 0.3
6^e^	OEGMA_950_	1	320	0.1		4.6 ± 0.3
7^e^	OEGMA_950_	1	320	0.32		16 ± 2

a Conditions: Monomer 20 vol % in DMEM with different amounts of DMSO, irradiated for 90 min under red light (λ_max_ = 625 nm, intensity 4 mW cm^–2^).

b OEGMA_500_ and OEGMA_950_ correspond to OEGMA macromonomers with *M_n_* ∼ 500 and ∼950 g mol^–1^, respectively.

c Measured by VASE.

d [MB^+^]:[PMDETA] = 1:24.

e Monomer 10 vol %.

Cytocompatibility of red light-mediated SI-bioATRP was demonstrated by incubating osteosarcoma cells (U2OS cell line) for 1 h with a polymerization mixture comprising 10 vol % OEGMA_950_ in DMEM, 1 vol % DMSO, and [Hb] = 320 μM, [MB^+^] = 0.32 mM. Additionally, cells were incubated with the same polymerization mixture and irradiated with red light (λ_max_ = 625 nm, intensity 4 mW cm^–2^) for 1 h. Notably, in both cases, cell viability remained >97% and comparable to the viability measured on the control populations (Figure 8a,b–d).

**Figure 8:**
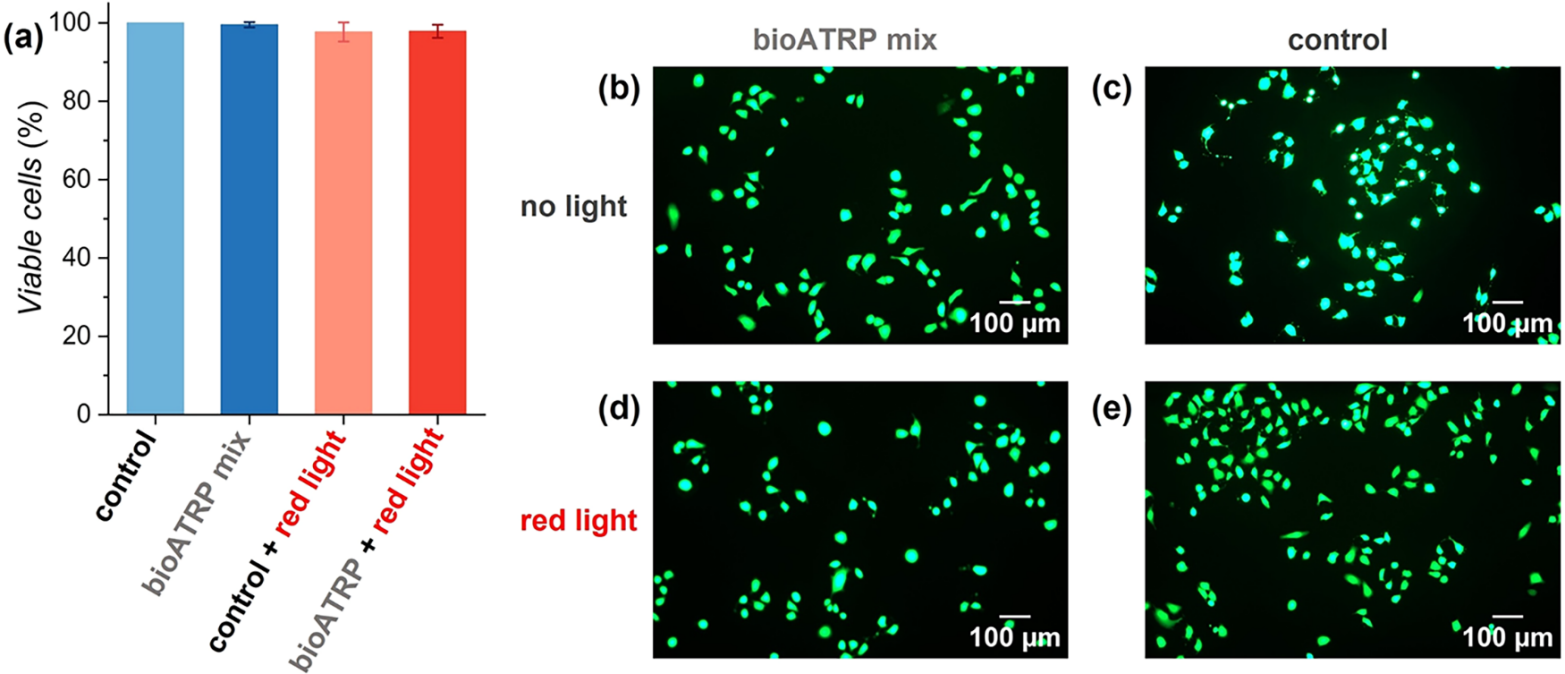
(a) Cell viability estimated by live-dead staining on U2OS cell cultures subjected for 1 h to a polymerization mixture including 10 vol % OEGMA_950_ in DMEM, with 1 vol % DMSO and [Hb] = 320 μM, [MB^+^] = 0.32 mM, and the corresponding controls subjected to a DMEM solution, in the dark or irradiated with red light (λ_max_ = 625 nm, 4 mW cm^–2^). (b–d) Immunofluorescence micrographs displaying U2OS cells stained with calcein-AM (green), after 1 h exposure to bioATRP mixture comprising 10 vol % OEGMA_950_ in DMEM, 1 vol % DMSO, and [Hb] = 320 μM, [MB^+^] = 0.32 mM, without (a) and with (c) simultaneous red light irradiation (λ_max_ = 625 nm, 4 mW cm^–2^); DMEM solution without (b) and with (d) simultaneous red light irradiation.

## Conclusions

In summary, we demonstrated that red light-mediated SI-bioATRP enables the controlled growth of polymer brushes in open air by harnessing the cooperative action of Hb and methylene blue/red light irradiation as an enzymatic-photochemical catalytic pair. MB^+^ coupled with red light rapidly generates Hb­(Fe^II^) activators in situ while simultaneously consuming dissolved oxygen, thereby maintaining the activation-deactivation equilibrium required for controlled radical polymerization under aqueous and mild conditions. Control over the grafting-from polymerization arises from the spontaneous adsorption of Hb onto initiator- and brush-decorated interfaces, which sustains reversible activation-deactivation cycles—otherwise inaccessible within homogeneous solution. Beyond enabling the synthesis of thick polymer brushes of diverse composition, this cytocompatible and oxygen-tolerant process establishes a general strategy for conducting surface-initiated controlled radical polymerizations under biologically relevant conditions. It thus represents an important step toward integrating polymer growth processes with living systems and contributes in expanding enzyme-assisted photopolymerization toward biofabrication and the development of advanced biomaterials.

## Supplementary Material



## Notes

Materials and experimental methods, electrochemical characterization, SEC analysis, and AFM characterization (PDF)
