# Supplementary material for: Cytocompatible Biocatalyzed Surface-Initiated PhotoATRP Mediated by Red Light Irradiation in Open Air
Source: JACS Au. 2026 Apr 29;6(5):3067–76. doi: 10.1021/jacsau.6c00580 (PMC13213514; doi:10.1021/jacsau.6c00580)
Supplement: Supplementary file 1 [file au6c00580_si_001.pdf]

SUPPORTING INFORMATION

Cytocompatible Biocatalyzed Surface-Initiated PhotoATRP Mediated by Red-Light Irradiation in Open Air

Yuwen Zhang,[a] Elena Avanzini,[a] Martina Ferrara,[a] Rebecca Busetto,[b] Veronica Torresan,[b] Alessandro Gandin,[b] Giovanna Brusatin,[b] Cristian Pezzato,[a] Francesca Lorandi,[a] Edmondo M. Benetti[a]**

[a] Laboratory for Macromolecular and Organic Chemistry, Department of Chemical Sciences, University of Padova, via Marzolo 1, 35131 Padova, Italy.

[b] Department of Industrial Engineering, University of Padova, via Marzolo 9, 35131 Padova, Italy.

1. Materials

Hemoglobin from bovine blood (Sigma-Aldrich), ethanol (absolute for analysis, Supelco), ultrapure water (Millipore Milli-Q grade), anhydrous dichloromethane (DCM, ≥99.8%, Sigma-Aldrich), toluene (≥99.7%, Sigma-Aldrich), anhydrous dimethyl sulfoxide (anhydrous DMSO, ≥99.9%, Sigma-Aldrich), sulfuric acid (H_2SO_4, 95-97%, Sigma-Aldrich), hydrogen peroxide (H_2O_2, 35% in water, TCI), acetic acid (99.8%-100.5%, Sigma-Aldrich), hydrochloric acid (≥37%, Sigma-Aldrich), (3-aminopropyl)triethoxysilane (APTES, ≥98%, Sigma-Aldrich), α-bromoisobutyryl bromide (BiBB, 98%, Sigma-Aldrich), triethylamine (TEA, ≥99.5%, Sigma-Aldrich), tin (II) 2-ethylhexanoate ($Sn(EH)_2$, ≥98%, Sigma-Aldrich), methylene blue hydrate (MB^+, ≥95%, Sigma-Aldrich), N,N,N′,N″,N″-pentamethyldiethylenetriamine (PMDETA, 98%, Sigma-Aldrich), anhydrous sodium acetate (EMSURE® ACS), Nafion solution (Ion Power), carbon black (Sigma-Aldrich), 2-hydroxyethyl 2-bromoisobutyrate (HEBiB, Sigma-Aldrich), (+)-Sodium L-ascorbate (NaAsc, Sigma-Aldrich), Tris base (≥99.8%, Sigma-Aldrich), cetyltrimethylammonium bromide (CTAB, Sigma-Aldrich), basic alumina (Sigma-Aldrich).

Oligo(ethylene glycol) methyl ether methacrylate (OEGMA$_{500}$, M_n ~ 500 g mol-1, Sigma-Aldrich), oligo(ethylene glycol) methyl ether acrylate (OEGA, M_n ~ 480 g mol-1, Sigma-Aldrich), 3-sulfopropyl methacrylate (SPMA, 98%, Sigma-Aldrich), 2-methacryloyloxyethyl phosphorylcholine (MPC, 97%, Sigma-Aldrich), *N*-isopropylacrylamide (NIPAM, >98%, TCI). 2-(methacryloyloxy)ethyl]trimethylammonium chloride solution (METAC, 75 wt. %, Sigma-Aldrich).

2. Methods

Electrochemical studies. Electrochemical measurements were performed using Metrohm VIONIC potentiostat/glavanostat (with INTELLO 1.4 software), in a standard three-electrode electrochemical cell. A glassy carbon (GC) electrode (2 mm diameter) was used as the working electrode. The GC surface was cleaned before each experiment, by polishing with 1 μm, 0.3 μm, and 0.05 μm alumina slurry, and ultrasonically rinsed in ethanol for 5 minutes after each polishing step. The reference electrode was a saturated calomel electrode (SCE), while the counter electrode was a Pt wire.

The Hb–CB/GC electrode was prepared as follows. About 4 mg carbon black powders were dispersed thoroughly in 5 mL of an aqueous solution containing 0.1 wt% cetyltrimethylammonium bromide (CTAB), which serves as a dispersant to prevent the CB powders aggregation. The dispersion was sonicated for 30 min. Then, 4 mg Hb was added to the suspension and stirred for 10 h at 4 °C. Then, 2 μL of the mixture was cast onto the surface of the glassy carbon electrode and dried at ambient temperature. Finally, 1 μL of Nafion solution was cast on the surface of the Hb–CB/GC electrode. After the solvent was evaporated, the Hb–CB/ GC electrode was obtained. If it was not used immediately, the electrode was stored at 4 °C in a refrigerator.

Variable-angle spectroscopic ellipsometry (VASE). The dry thickness of polymer brushes (T_{dry}) was measured by using a M-2000V variable-angle spectroscopic ellipsometer (VASE, J.A. Woollam Co.) equipped with a 50 W Quartz Tungsten

Halogen lamp (FQTH-100). Amplitude (Ψ) and phase (Δ) were acquired at an angle of incidence of 65° as a function of wavelength (370–1000 nm). The fitting of raw data was analyzed using WVASE32 software, employing a layered model with bulk dielectric functions for Si and SiO_2. The polymer brush layers were characterized using a Cauchy model defined as $n=A+B\lambda^{-2}$, where A and B were set to 1.45 and 0.01 (values for transparent organic films), while λ is the wavelength, and n represents the refractive index. The thickness of each sample was measured on three spots to calculate the average T_{dry} values and standard deviations.

UV-Vis Spectroscopy. UV-Vis spectra were collected using an Agilent Cary 60 spectrometer equipped with an 18-cell holder coupled to a Huber thermostat, using Suprasil quartz cuvettes (114-QS) from Hellma Analytics. Samples for the determination of the reduction rate of Hb(Fe^{III}) were prepared by diluting 4 times a typical polymerization mixture (described in Section 3). Scans were performed in the wavelength range from 300 to 550 nm with an interval of 1 nm.

^{1}H NMR. ^{1}HNMR spectra were recorded using a Bruker Advanced III 400 MHz spectrometer at room temperature and using D_2O as solvent.

Size exclusion chromatography (SEC). SEC was performed by using a Viscotek gel permeation chromatography (GPC) system (Malvern, Worcs, U.K.) equipped with a pump and a degasser (GPCmax VE2001, 1.0 mL min^{-1} flow rate), a detector module (Viscotek 302 TDA), and two columns (2×Agilent GRAM columns, dimensions 8 mm × 300 mm, particle size 10 µm) using DMF with 10 mM LiBr as eluent. Each sample was prepared by dissolving the polymer at a defined concentration of 1 mg mL^{-1} in DMF containing 10 mM LiBr. Poly (methyl methacrylate) standards with molecular weight ranging from 2500 to 212000 Da were used for calibration.

Isothermal titration calorimetry (ITC). ITC measurements were performed using Microcal PEAQ-ITC at 25 °C. For each titration, a 600 µM hemoglobin (Hb) solution was used as the titrant and loaded into the syringe (70 µL). The sample cell was filled with either a SiO_2-*g*-POEGMA nanoparticle dispersion (synthetic procedure in Section 3) or free POEGMA solution.

The organic content of the SiO_2-*g*-POEGMA nanoparticles (1 mg/mL), determined by thermogravimetric analysis (TGA) was 64.3%. M_{brush} was calculated using the following equation:

$$\sigma = \frac{\frac{(\%w/w)_{brush}}{(\%w/w)_{SiO2}} \rho_{SiO2} V_{SiO2} N_A}{M_{brush} A_{SiO2}} \quad (1)$$

where σ is the grafting density, was calculated to be ~0.6 chains nm^2, $(\% \text{ w/w})_{\text{brush}}$ is the percentage of mass loss in TGA for the organic fraction, $(\% \text{ w/w})_{\text{SiO2}}$ is the residual mass percentage of the inorganic fraction in TGA. N_{A} is the Avogadro constant, ρ_{SiO2} is the density of silica, V_{SiO2} is the volume and A_{SiO2} is the area of the SiO_2 core calculated from the diameter of the nanoparticles measured by TEM. Thus, the molar mass of the polymer brush was ~300 kDa. This corresponds to a polymer brush concentration of 1.8 µM in the nanoparticle solution. The concentration of free POEGMA was matched to that grafted on the nanoparticles. All samples were freshly prepared and filtered through a 0.22 µm cellulose acetate filter. During the titration, 2 µL of titrant was injected every time to the sample cell via a rotating stirrer syringe (750 rpm), for a total of 19 injections. The first injection of 0.4 µL is routinely removed from the analysis to avoid artifacts. The data processing was done using the MicroCal PEAQ-ITC analysis software. A "One Set of Sites" binding model was employed to best reflect the nature of interaction with polymer brush shell. Blank titrations were carried out in order to estimate heat changes arising due to dilution of the ligand, and the heat changes were subtracted from the changes observed in the main titration.

Quartz crystal microbalance with dissipation (QCM-D). QCM-D sensors (QSX 303 SiO_2, Biolin Scientific) were cleaned by immersion in freshly prepared piranha solution for 30 seconds. After rinsing with MilliQ and EtOH, sensors were dried with a stream

of N_2. The cleaned QCM-D chips were functionalized with an alkyl halide initiator following the same protocol applied to silica-coated wafers (Section 3).

For experiments involving the polymerization mixture, the functionalized sensors were first stabilized in Milli-Q water at ambient temperature until a stable baseline was recorded (30 min). The Milli-Q water was then replaced with a control polymerization mixture without Hb, and it was monitored for 30 min. Subsequently, this was replaced with a standard polymerization mixture containing Hb to monitor its binding for a further 30 min. To assess binding stability, the sensors were washed sequentially with the control polymerization mixture (without Hb) for 30 min and finally with Milli-Q water for another 30 minutes. Frequency shifts (ΔF) were recorded using the 7th, 9th, and 11th overtones.

Cyclic voltammetry of the catalytic system. For studying the behavior of MB^+ upon addition of Hb, the electrochemical cell was assembled with the electrodes described above, utilizing the bare GC, and maintained under an inert atmosphere by continuous Ar flow. The MB^+ and acetate buffer were added in the cell and CVs were recorded at different scan rates. The Hb solutions (50 μM and 75 μM) were subsequently added and CVs were recorded after every addition. For analyzing the response of Hb upon addition of HEBiB and MB^+, CV were recorded in acetate buffer at pH 6 and 7 across a range of scan rates, under Ar flow.

Functionalization of SiO_x substrate with ATRP initiator layer. Silicon substrates (1 cm × 2 cm) were first cleaned by immersion in piranha solution (3:1 mixture (v/v) of H_2SO_4 and H_2O_2) for 1 h. The substrates were then rinsed thoroughly with ultrapure water and ethanol, followed by drying under a stream of N_2. Subsequently, the cleaned substrates were placed in a desiccator and functionalized with (3-aminopropyl)triethoxysilane (APTES) via vapor-phase deposition under vacuum for 3 hours. The resulting functionalized substrates were washed sequentially with ultrapure water, ethanol, toluene, and then dried under N_2. α-Bromoisobutyryl bromide (BiBB, 0.2 mL), triethylamine (TEA, 0.2 mL) and dry dichloromethane (DCM, 20 mL) were

added to an Erlenmeyer flask containing the APTES-bearing substrates and let react for 2 h. Subsequently, the ATRP initiator-functionalized substrates were washed with DCM and ethanol and dried under N_2.

Typical procedure for the preparation of the polymerization mixture. First, the stock solution of 25 mM MB^+ in acetate buffer was prepared. A representative polymerization mixture was then prepared by mixing acetate buffer (pH 5, 3.45 mL), OEGMA (M_n ~500 Da, 1 mL), DMSO (500 μL), PMDETA (6.3 μL), hemoglobin (5.2 mg), and an aliquot of the MB^+ stock solution (50 μL). The resulting mixture had a final pH of approximately 6, as measured by a pH meter.

Red-light mediated SI-bioATRP under open air. An initiator-functionalized substrate was placed on a glass Petri dish (with a diameter of 5.7 cm), which was filled with 5 mL of polymerization mixture, paying attention to uniformly covering the substrate. The red-light source was positioned above the Petri dish at a distance of 25.5 cm to obtain a light intensity on the substrate of 4 mW cm^{-2}. For SI-bioATRP under different light wavelengths, the same light source was employed and set to the desired wavelength (UV: λ_{max} = 365 nm, blue: λ_{max} = 420 nm or λ_{max} = 475 nm, green/yellow: λ_{max} = 565 nm, NIR: λ_{max} = 780 nm). The distance of the source from the substrate was regulated to keep the light intensity on top of the substrate constant at 4 mW cm^{-2}. After polymerization, the light was turned off and the substrates were thoroughly washed with ultrapure water and ethanol, followed by drying under a stream of N_2.

In the case of multiple reinitiation experiments, each polymerization time was set to 90 min. After each polymerization, the substrates were removed from the polymerization mixture and washed extensively with ultrapure water and ethanol and dried under a stream of N_2. Subsequently, the dry thickness of the polymer films was measured by VASE before incubation in a freshly prepared polymerization mixture for the subsequent cycle.

NaAsc mediated SI-bioATRP in a deoxygenated environment. A typical polymerization was conducted as follows. OEGMA (440 mg, 440 mM), hemoglobin (Hb, 2 mg, 16 μM), and acetate buffer (0.1 M, 1.1 mL) were combined in a vial with an initiator-functionalized substrate. In a separate vial, a fresh aqueous stock solution of sodium ascorbate (NaAsc, 17.6 mM) was prepared. Both solutions were thoroughly deoxygenated by purging with Ar for 30 min. Subsequently, 0.5 mL of the deoxygenated NaAsc solution was transferred into the polymerization mixture under inert atmosphere. The reaction was conducted at room temperature for the desired duration.

For reinitiation experiments, each polymerization cycle was set to 90 min. After each cycle, the substrates were removed, washed extensively with ultrapure water and ethanol, and dried under a stream of N_2. The dry film thickness was measured by VASE. The substrates were then reimmersed in a freshly prepared and deoxygenated polymerization mixture to initiate the next 90-min cycle. This cycle was repeated several times.

Red-light mediated bioATRP in solution. A vial was filled with 5 mL of polymerization mixture and 3.1 μL of HEBiB (target degree of polymerization = 100). The solution was deoxygenated by Ar for 30 min. The red-light source was positioned on the left of the vial at a distance of 25.5 cm to obtain a light intensity on the wall of the vial of 4 mW cm^{-2}. The reaction was stirred at room temperature, and 0.5 mL samples were removed for analytical purposes at periodic intervals. The analytical sample was dried under compressed air, and then mixed with 1 mL DMF with 10 mM LiBr. The sample was filtered through a small plug of neutral aluminum oxide and then analyzed by GPC. The remaining sample was analyzed by ^{1}H NMR.

Preparation of SiO_2-*g*-POEGMA nanoparticle. 200 nm particles were added in a beaker containing a piranha solution (H_2SO_4 and H_2O_2, 3:1 v/v). After 30 min the piranha solution was diluted with MilliQ water and immersed in an ice bath letting the particles to sediment. After 2h the diluted piranha solution was removed using a Pasteur

pipette and added again with MilliQ water, repeating the same procedure until the solution was no more acidic (checked with a litmus paper). The particles were then transferred in a 15 mL falcon and washed one more time with water and 2 times with EtOH (~10 mL). The three centrifuges were performed at 6000 rpm for 5 min. After this procedure the activated particles were let under vacuum overnight to dry.

The activated SiO_2 particles (100 mg) were suspended in a vial with dry toluene (5 mL) under magnetic stirring. Under stirring, APTES (100 µL, 427 mmol) was added. The sealed vial was put under vigorous stirring for 14 h at 100 °C. The suspension was transferred into a 15 mL vial and toluene was added. The mixture was centrifuged for 5 min at 6000 rpm and the supernatant gently removed. This operation was repeated one more time with toluene and 3 times with EtOH. The particles where then let to dry overnight under vacuum.

The APTES-functionalized particles obtained from the previous step were put in a clean vial with 5 mL of dry DCM. The solution was degassed for 30 min. While under stirring, TEA (119 µL, 854 µmol) was added, followed by the dropwise addition of BiBB (106 µL, 854 µmol). The reaction was stirred under inert atmosphere at room temperature for 14 h. Following the same procedure, the suspension was transferred to a 15 mL falcon and washed 2 times with DCM and 3 times with EtOH (about 10 mL each wash) and then let to dry overnight.

For the grafting of polymer brushes onto the particles, the following procedure was used: in a 10 mL round-bottom flask, 2.5 mL of filtered OEGMA (M_n ~300 Da, 8.7 mmol) were added to 2.4 mL of DMSO. As catalyst, 1 mM $CuBr_2$ was used with TPMA as ligand ([$CuBr_2$]:[L], 1:3). The copper catalyst was added from a stock solution (0.02 M) prepared in DMSO. 30 mg of initiator bearing NPs were suspended in the polymerization mixture under vigorous stirring. The flask was sealed and the solution degassed with argon for 30 min. After that, 0.5 mM of NaAsc was added from a degassed aqueous stock solution, reaching a 2 vol% of water in the polymerization mixture. The reaction was let under vigorous stirring and argon overpressure for 90 minutes.

The brushes functionalized NPs were transferred in a 15 mL falcon and purified with several washes before use (2 washes with DMSO, 2 with EtOH, 1 with MilliQ water and EDTA and 2 more with EtOH). Finally, they were dried under vacuum overnight.

Synthesis of POEGMA by activator regenerated by electron transfer (ARGET) ATRP. POEGMA was synthesized by ARGET-ATRP of 50:1:0.05:0.07:0.05 $OEGMA_{500}$:HEBiB:$CuBr_2$:TPMA:$Sn(EH)_2$ in DMF, [OEGMA] = 1 M. $CuBr_2$ (11.17 mg) and TPMA (20.33 mg) were dissolved in 5 mL of DMF, and 0.5 mL of this solution were added to the polymerization solution. For the synthesis of POEGMA, OEGMA (2.5 g, 5 mmol), HEBiB (14 μL, 0.1 mmol), $CuBr_2$ (1.12 mg, $5 \cdot 10^{-3}$ mmol), TPMA (2.03 mg, $7 \cdot 10^{-3}$ mmol), and DMF (1.4 mL) were poured into a Schleck flask 15 mL and degassed with Ar for 30 mins. Then, in a 25 mL round bottom flask, $Sn(EH)_2$ (2.03 mg, $5 \cdot 10^{-3}$ mmol) was dissolved in anisole and degassed with Ar for 30 min. Finally, 0.5 mL of the solution of reducing agent was added to the solution containing the monomer. The polymerization was carried out at room temperature for 12 h and stopped by exposure to air. The final product was obtained after purification by dialysis (cut off 1 kDa) in MeOH for two days, evaporating under reduced pressure, and freeze drying.

Cytocompatibility tests of red light-mediated SI-bioATRP. Osteosarcoma cells (U2OS) were cultured in DMEM supplemented with 10% V/V FBS, 1% V/V glutamine and 1% V/V Penicillin/Streptomycin. Cell viability was evaluated using a live/dead assay (Live/Dead Viability/Cytotoxicity Kit, for mammalian cells, Invitrogen) with calcein-AM, to detect live cells, and ethidium homodimer-1 (EthD-1), to detect dead cells. U2OS were seeded at a density of 4000 cells/cm^2 and monitored for 1 day. Then the cell culture medium was replaced by 500 μL bioATRP mixture, which comprises 10 vol% $OEGMA_{950}$ in DMEM, with 1 vol% DMSO and 320 μM Hb and 0.32 mM MB^+, followed by incubation for 60 min with or without red light irradiation. A control group was treated with complete culture medium alone with or without red light irradiation. After treatment, the solution was replaced with 1X HBSS (Hanks' Balanced

Salt Solution) containing 2 μM calcein-AM and 4 μM EthD-1, and the cells were incubated for 60 min at 37 °C under 5% CO_2 in the dark. Finally, the samples were imaged using a widefield fluorescence microscope.

3. Characterization of MB^+/Hb and NaAsc/Hb redox behavior

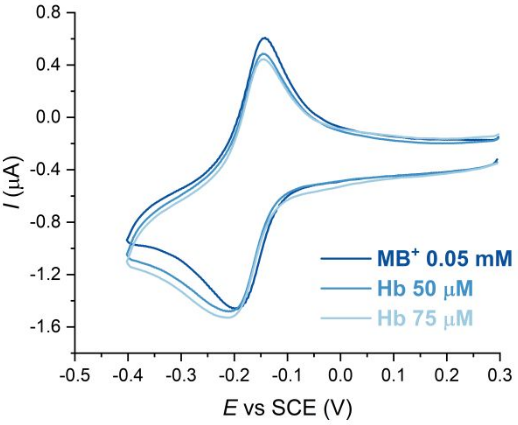

Figure S1. CV of 0.25 mM MB^+ recorded on a GC WE at a scan rate of 50 mV s^{-1}, in acetate buffer at pH 6.

Hb exhibits a sluggish electron transfer with electrode surfaces because the heme groups are rather buried within peptide chains. Therefore, CVs of Hb were recorded by immobilizing the protein onto the surface of a GC electrode, together with carbon black, a cationic surfactant, and Nafion as binder, following a reported procedure.[40] CV of the Hb@GC electrode in acetate buffer at pH 6 showed a quasi-reversible behavior, with similar anodic and cathodic currents and a peak separation of ~100 mV. The half-wave potential was $E_{1/2} = (E_{p,a} + E_{p,c})/2 = -0.33$ V *vs* SCE (with $E_{p,a}$ and $E_{p,c}$ the anodic and cathodic peak currents, respectively), in agreement with literature values,[40] which is typically attributed to a non-denaturated Hb. CVs at various scan rates (FigureS2a) revealed a direct proportionality between the cathodic peak current and the scan rate (FigureS2b), indicative of a redox process involving species adsorbed onto the electrode surface.

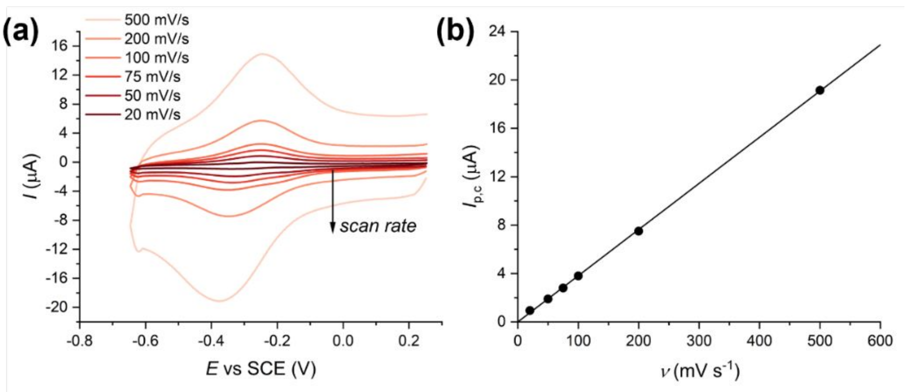

Figure S2. (a) CVs of the Hb@GC electrode in acetate buffer at pH 6 with varying the scan rate from 20 to 500 mV s^{-1}. (b) The plot of cathodic peak current vs. scan rate showing a linear proportionality between $I_{p,c}$ and v.

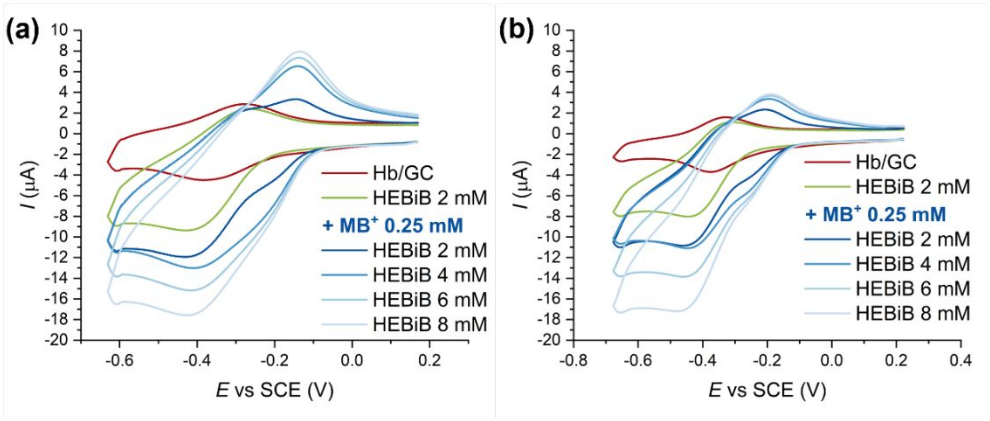

Figure S3. CVs of the Hb@GC electrode in acetate buffer at pH (a) 6 and (b) 7, at a scan rate of 100 mV s^{-1}, in the absence and presence of 0.25 mM MB^{+} and increasing amounts of HEBiB.

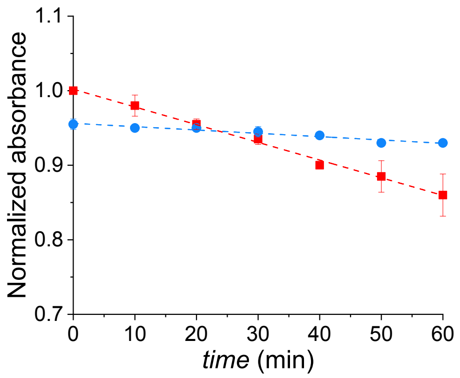

Figure S4. Variation of the absorbance of Hb at λ_{max} = 404 nm over time upon addition of MB^{+} under red-light irradiation (λ_{max} = 625 mM, intensity = 4 mW cm^{-2}) in open air (red squares), and upon addition of NaAsc in a deoxygenated cuvette (blue dots). Conditions: acetate buffer at pH 6, 0.108 M OEGMA,

[OEGMA]:[MB^+]:[Hb]:[PMDETA] = 27000:15.6:1:375 with 2.5 vol% DMSO, or [OEGMA]:[NaAsc]:[Hb] = 27000:275:1.

4. Control experiments for red-light mediated SI-bioATRP

Table S1. Influence of the system's components on POEGMA brush growth by red-light mediated SI-bioATRP in open air.[a]

Entry	Hb (μM)	MB^+ (mM)	PMDETA (mM)	Light intensity (mW cm^{-2})	T_{dry} (nm) [b]
1	-	0.25	6	4	1.8 ± 0.1
2	16	-	6	4	6.6 ± 0.1
3	16	0.25	-	4	20.8[c]
4	16	0.25	1	4	25.8 ± 0.8
5	16	0.25	6	-	6.1 ± 0.1

[a]Conditions: OEGMA 20 vol%, DMSO 10 vol%, acetate buffer 70 vol% (pH 6), [MB^+]:[Hb]:[PMDETA] = 25:1.6:600, [Hb] = 16 μM, irradiated for 90 min under red light (λ_{max} = 625 nm, 4 mW cm^{-2}). [b]Measured by VASE. [c]The substrates are inhomogeneous.

5. Red-light mediated bioATRP in solution

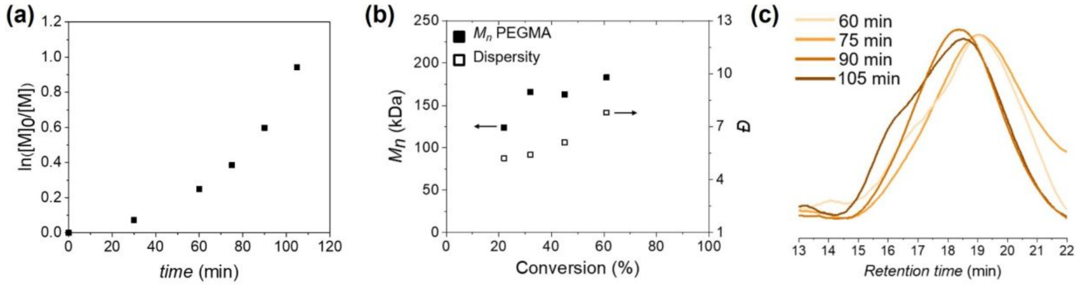

Figure S5. Red-light mediated bioATRP in solution. First-order kinetic plot (a), evolution of molecular weight and molecular weight distribution with monomer conversion (b), and SEC traces evolution with time (c). Conditions: Red-light mediated bioATRP of OEGMA 20 vol% in acetate buffer with DMSO 10 vol%, pH 7, [Hb] = 16 μM, [OEGMA]:[HEBiB] = 100:1, [MB^+]:[Hb]:[PMDETA] = 15.6:1:375, λ_{max} = 625 mM, intensity = 4 mW cm^{-2}.

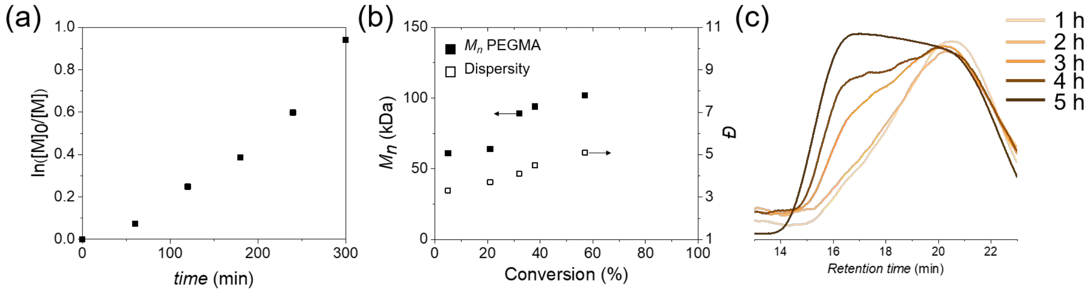

Figure S6. Red-light mediated bioATRP in solution. First-order kinetic plot (a), evolution of molecular weight and molecular weight distribution with monomer conversion (b), and SEC traces evolution with time (c). Conditions: Red-light mediated bioATRP of OEGMA 20 vol% in acetate buffer with DMSO 10 vol%, pH 7, [Hb] = 100 μM, [OEGMA]:[HEBiB] = 100:1, [MB^+]:[Hb]:[PMDETA] = 15.6:1:375, λ_{max} = 625 nm, intensity = 4 mW cm^{-2}.

6. pH effect on red-light mediated bioATRP in solution

Table S2. Red-light mediated bioATRP in solution at different pH[a].

Entry	Buffer	Final pH	Conversion
1	Acetate buffer	4	0%
2	Acetate buffer	5	0%
3	Acetate buffer	6	0%
4	Tris-HCl	7	>99%
5	Tris-HCl[b]	7	~85%
6	Tris-HCl	8	>99%
7	Tris-HCl	9	>99%

[a]Conditions: OEGMA 20 vol%, DMSO 10 vol%, buffer 70 vol%, [MB^+]:[Hb]:[PMDETA] = 25:1.6:600, [Hb] = 16 μM, The solution was degassed with Ar for 30 min and then irradiated for 120 min at a fixed light intensity of 4 mW cm^{-2}; [b] same as [a]conditions but the polymerization was conducted in open air.

7. Interaction of Hb with POEGMA.

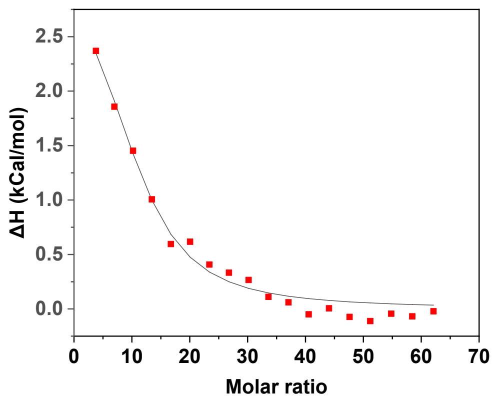

Figure S7. Fitting of the ITC measurement performed on a dispersion of 0.07 nM SiNP-*g*-POEGMA subjected to 19 sequential injections of 2 μL aliquots of [Hb] = 600 μM in acetate buffer (Figure 5a in the manuscript). Heat rates were corrected for the heat of dilution. The x-axis shows the ratio of [Hb]:[POEGMA].

The fitting in Figure S6 enable to quantify the thermodynamic parameters for the interaction of Hb with POEGMA grafts on the surface of NPs. The number of binding sites (n) and the dissociation constant (K_D) were n = 10.5 ± 0.6, and K_D = (3.1 ± 1.0) × 10^{-6} M. Additionally, ΔH = -12.13 ± 1.3 kJ mol^{-1}, ΔG = -31.46 kJ mol^{-1}, and ΔS = 146 J K^{-1}.

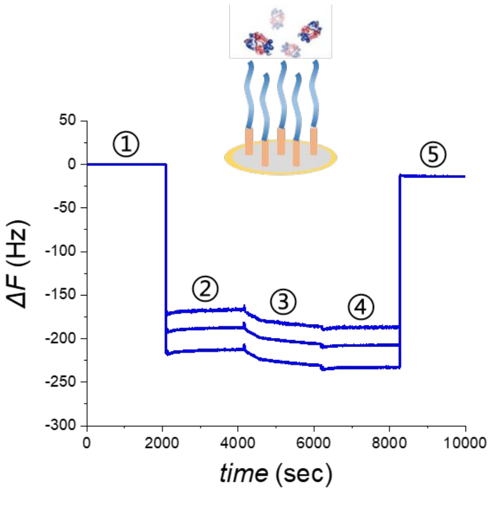

Figure S8. QCM-D sensogram displaying the variation of ΔF (three different overtones: f-5th, f-7th, and f-9th) for the POEGMA brush-functionalized sensor. Initially, acetate buffer was injected (1), followed by exposure to the polymerization mixture without Hb (2). Then, the complete polymerization mixture including Hb (3) was injected, followed by rinsing with the polymerization mixture lacking Hb (4) and finally rinsing with buffer (5).

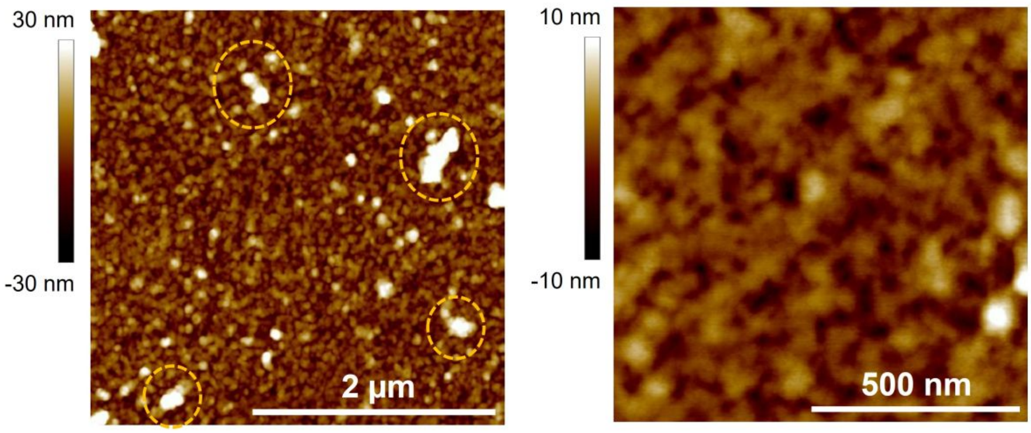

Figure S9. High resolution AFM height micrographs recorded in air using tapping mode method depicting POEGMA brushes synthesized by red light-mediated SI-bioATRP. The clusters highlighted by the orange dashed circles are associated with residual aggregates of Hb that remained on the brush following rinsing procedures.

8. SI-bioATRP under light irradiation at different wavelengths.

Table S3. SI-bioATRP of OEGMA in open air under light irradiation at different wavelengths.[a]

Entry	Light	λ_{max} (nm)	T_{dry} (nm)[b]
1	UV	365	13.1 ± 0.6
2	Blue	420	12.1 ± 0.5
3	Blue	475	28.1 ± 3.6
4	Green/Yellow	565	20.5 ± 0.5
5	NIR	780	5.2 ± 0.1

[a]Conditions: OEGMA 20 vol%, DMSO 10 vol%, acetate buffer 70 vol% (pH 6), [MB^+]:[Hb]:[PMDETA] = 25:1.6:600, [Hb] = 16 µM, irradiated for 90 min at a fixed light intensity of 4 mW cm^{-2}. [b]Measured by VASE.
